# Association Between Free Sugar Intake and Risk of Dyslipidemia in Chinese Adults—Evidence from the Chinese Food Consumption Survey and the China Health and Nutrition Survey

**DOI:** 10.3390/foods15162945

**Published:** 2026-08-21

**Authors:** Feng Pan, Huijun Wang, Zhihong Wang, Bing Zhang, Chang Su, Jiguo Zhang, Xiaofang Jia, Wenwen Du, Hongru Jiang, Liusen Wang, Weiyi Li, Lixin Hao, Weifeng Mao, Tongwei Zhang, Fanglei Zhao, Jianwen Li, Gangqiang Ding

**Affiliations:** 1 China National Center for Food Safety Risk Assessment, Beijing 100022, China; panfeng@cfsa.net.cn (F.P.); maoweifeng@cfsa.net.cn (W.M.); zhangtongwei@cfsa.net.cn (T.Z.); zhaofanglei@cfsa.net.cn (F.Z.); 2 National Institute for Nutrition and Health, Chinese Center for Disease Control and Prevention, Beijing 100050, China; wanghj@ninh.chinacdc.cn (H.W.); wangzh@ninh.chinacdc.cn (Z.W.); zhangbing@chinacdc.cn (B.Z.); suchang@ninh.chinacdc.cn (C.S.); zhangjg@ninh.chinacdc.cn (J.Z.); jiaxf@ninh.chinacdc.cn (X.J.); duww@ninh.chinacdc.cn (W.D.); jianghr@ninh.chinacdc.cn (H.J.); wangls@ninh.chinacdc.cn (L.W.); liwy@ninh.chinacdc.cn (W.L.); haolx@ninh.chinacdc.cn (L.H.)

**Keywords:** free sugar intake, dyslipidemia, adults, Chinese Food Consumption Survey, China Health and Nutrition Survey

## Abstract

The overall prevalence of dyslipidemia among adults continues to rise. However, limited attention has been devoted to the association between free sugar intake and dyslipidemia. This study aimed to explore the association between free sugar intake and dyslipidemia, as well as its subtypes, in Chinese adults. A total of 38,656 adults from the Chinese Food Consumption Survey (CFCS) and 3974 from the China Health and Nutrition Survey (CHNS) were included in the final analysis. Dietary intake data were collected by 24 h dietary recalls and the weighing of household foods and condiments. For the CHNS, multivariable Cox proportional hazards regression models were used to examine the association between free sugar intake and the risk of dyslipidemia and its subtypes. Restricted cubic spline (RCS) regression was used to evaluate the nonlinear relationship between free sugar intake and dyslipidemia. Stratified analyses and interaction tests were performed across key covariates to explore potential effect modifiers of the association between free sugar intake and dyslipidemia risk. For the CFCS, the mean free sugar intake among Chinese adults was 6.4 g/d. For the CHNS, a total of 1052 individuals were diagnosed with dyslipidemia, corresponding to an incidence rate of 26.5% during a total of 24,531 person-years of follow-up (median follow-up, 6.0 years) in the CHNS cohort. After multivariable adjustment, the risk of dyslipidemia was increased by 38% with >20 g/d free sugar intake (HR = 1.38, 95% CI: 1.04–1.84, *p* for trend = 0.006), with <5 g/d free sugar intake as a reference. For different subtypes of dyslipidemia, with <5 g/d free sugar intake as a reference, the risk of hypo-HDL-cholesterolemia and hyper-LDL-cholesterolemia was increased by 91% (HR = 1.91, 95% CI: 1.34–2.74, *p* for trend < 0.001) and 52% (HR = 1.52, 95% CI: 1.01–2.29, *p* for trend = 0.004) with > 20g/d free sugar intake, respectively. Stratified analyses demonstrated that the impact of high free sugar intake on the development of hypo-HDL-cholesterolemia and hyper-LDL-cholesterolemia was significantly amplified among urban residents and those with insufficient physical activity. While linear dose–response associations were observed between free sugar intake and the risk of dyslipidemia (*p* for nonlinearity > 0.05), a nonlinear, inverted U-shaped association emerged for hyper-LDL-cholesterolemia (*p* for nonlinearity = 0.007). Our study demonstrates that free sugar intake levels in Chinese adults were relatively low compared to the recommended intake level (<50 g/d) in the Chinese Dietary Guidelines 2022. Nevertheless, increased free sugar intake was positively associated with a higher risk of incident dyslipidemia, as well as incident hypo-HDL-cholesterolemia and hyper-LDL-cholesterolemia. Targeted nutrition education is recommended to promote balanced dietary patterns and enhance overall dietary quality, thereby effectively reducing the risk of dyslipidemia.

## 1. Introduction

Dyslipidemia is defined as abnormal serum lipid profiles, including elevated total cholesterol (TC), triglycerides (TG), low-density lipoprotein cholesterol (LDL-C), and/or a reduction in high-density lipoprotein cholesterol (HDL-C) [1]. In recent decades, the prevalence of dyslipidemia has been increasing among the Chinese population. Data from the 2007–2010 Chinese Chronic Kidney Disease Epidemiology Survey showed that the prevalence of dyslipidemia among adults aged 18 years and older in China was 34.0%, with rates of 35.1% in urban areas and 26.3% in rural areas [2]. Meanwhile, results from the Report on Nutrition and Chronic Diseases Status of Chinese Residents (2020) revealed that in 2018, the prevalence of dyslipidemia among Chinese residents aged 18 and above was 35.6%, with rates of 36.5% in urban areas and 34.6% in rural areas [3]. The prevalence rates for hypercholesterolemia (elevated TC), hypertriglyceridemia (elevated TG), hypo-HDL-cholesterolemia (low HDL-C), and hyper-LDL-cholesterolemia (elevated LDL-C) were 8.2%, 18.4%, 20.9%, and 8.0%, respectively [3]. This situation has made dyslipidemia a major public health burden in China. Numerous epidemiological studies have established that dietary and lifestyle factors are key modifiable determinants of dyslipidemia onset and progression [4,5]. Dietary modifications can effectively prevent and delay the development of dyslipidemia. Consequently, investigating the link between dietary factors and dyslipidemia can provide a scientific foundation for the prevention and control of dyslipidemia and its related cardiovascular diseases.

Free sugars are defined by the World Health Organization (WHO) as monosaccharides (e.g., glucose and fructose) and disaccharides (e.g., sucrose) added to foods and beverages by manufacturers, cooks, or consumers, as well as sugars naturally present in honey, syrups, fruit juices, and fruit juice concentrates [6]. A growing body of research in recent years has shown that excessive consumption of free sugars is associated with an increased risk of overweight, obesity, type 2 diabetes, metabolic syndrome (MetS), and subsequent cardiovascular diseases [7,8,9,10]. Specifically, relevant studies have found an association between high levels of free sugar intake and the development of dyslipidemia. An analysis of data from the National Health and Nutrition Examination Survey (NHANES) in the United States (1999–2006), involving 6113 adults aged 18 years and older, examined the association between added sugars intake and dyslipidemia. The study demonstrated that as the percentage of energy derived from added sugar increased, adverse effects on blood lipid levels were observed, including a decrease in HDL-C and an increase in TC [11].

However, existing epidemiological evidence on the association between dietary sugar intake and dyslipidemia is predominantly derived from Western populations in the United States and Europe, while data from Asian populations—particularly Chinese adults—remain scarce. In 2017, the Chinese government issued the National Nutrition Plan (2017–2030), which explicitly incorporates a national sugar reduction initiative as a core public health priority [12]. Accordingly, the association between dietary sugar intake and chronic diseases has attracted growing research attention in the fields of nutrition and public health. Existing epidemiological studies have largely focused on the links between dietary sugar intake and overweight/obesity, type 2 diabetes, and cardiovascular disease. Dyslipidemia is a well-established key risk factor for atherosclerosis; however, population-based evidence regarding the relationship between free sugar intake and dyslipidemia remains limited in China. To address this gap, we conducted the present study to examine the longitudinal association between free sugar intake and dyslipidemia, with the aim of providing scientific evidence to support national sugar reduction strategies and reduce the population-level burden of cardiovascular disease. Therefore, we aimed to use data from the Chinese Food Consumption Survey (CFCS) and the China Health and Nutrition Survey (CHNS) to describe free sugar intake levels and evaluate the associations between dietary free sugar intake and the risk of incident dyslipidemia and its subtypes in Chinese adults. Our findings will provide evidence to support the development of targeted nutrition policies and dietary guidelines to prevent and control dyslipidemia in China.

## 2. Materials and Methods

### 2.1. Study Design and Population

Data for this study were obtained from the CFCS and the CHNS. The CFCS is a nationally representative cross-sectional study led by the China National Center for Food Safety Risk Assessment. Conducted from 2017 to 2020 across 126 survey sites in 23 provinces, the CFCS aimed to characterize food consumption patterns and assess dietary intake, nutrient status, and food safety risks in the Chinese population. The survey employed a multistage stratified cluster random sampling design to collect data on dietary intake, food processing practices, food contact materials, demographic and geographic characteristics, socioeconomic status, and physical examination findings [13]. The CHNS is a prospective, ongoing and longitudinal cohort study in China. The survey was initiated in 1989, with follow-ups conducted every 2–4 years in 1991, 1993, 1997, 2000, 2004, 2006, 2009, 2011, 2015, and 2018. The CHNS used a multistage random cluster sampling method to collect demographic geography, community conditions, economic activity, diet, and health information in 15 provinces to understand the impact of social and economic changes on the population’s health and nutritional status. Detailed information regarding the survey’s background, objectives, design, and methodology can be found elsewhere [14,15]. The CHNS collected and tested blood samples during three waves in 2009, 2015, and 2018. Accordingly, this study utilized data from these three waves for analysis in the CHNS.

**Figure 1:**
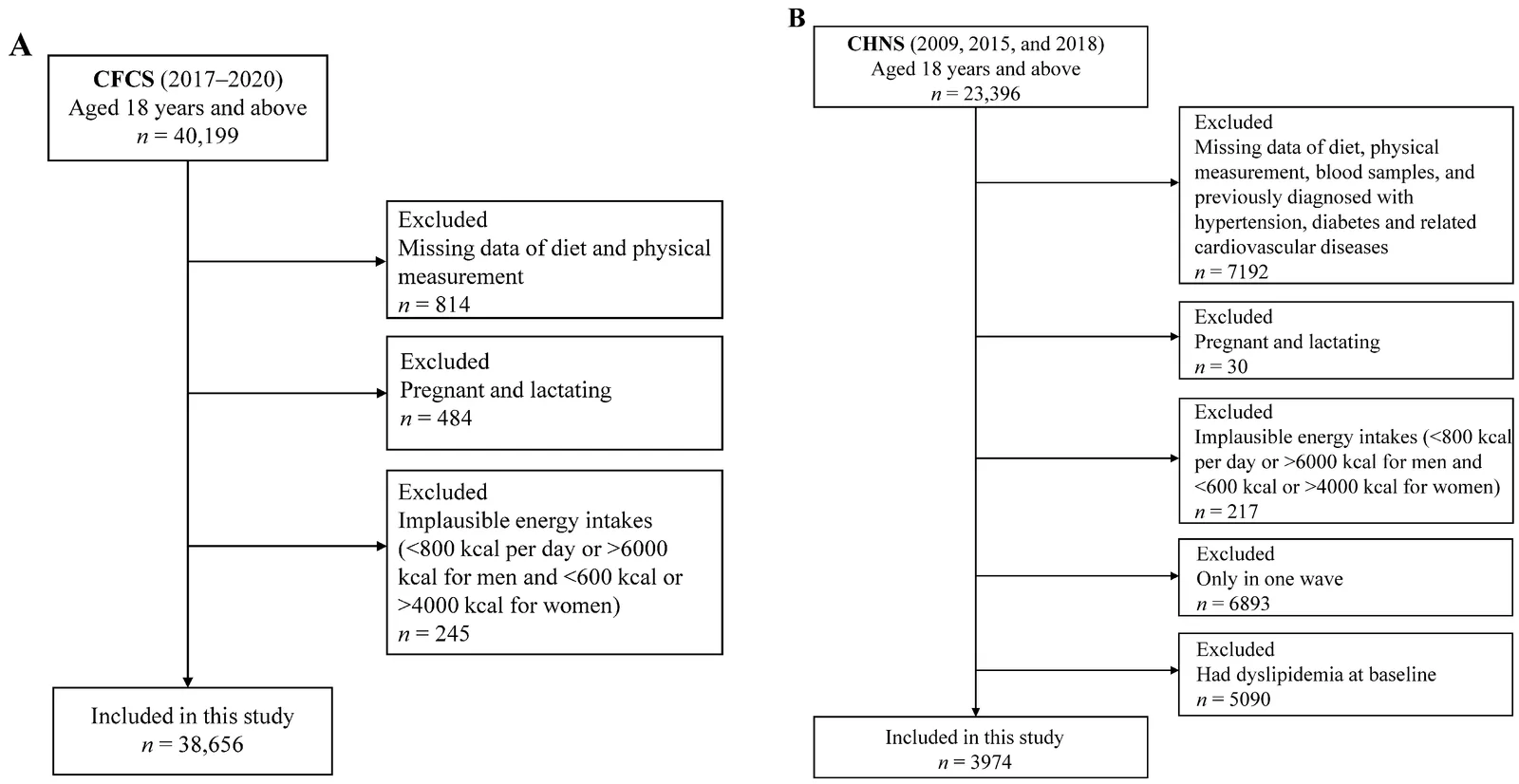
Flowchart of the participants from the CFCS (**A**) and the CHNS (**B**) included in this study.

For both the CFCS and the CHNS, we applied strict inclusion and exclusion criteria to ensure the robustness of the analysis. Adults aged 18 years and older who had complete dietary survey data, as well as data on socioeconomic status, lifestyle, and anthropometric measurements, were included in both the CFCS and the CHNS. For the CFCS, a total of 814 participants were excluded due to missing dietary and physical measurement data. We excluded 484 pregnant or lactating women and 245 participants reporting implausible total energy intake (Men: <800 kcal/day or >6000 kcal/day; Women: <600 kcal/day or >4000 kcal/day). Finally, a total of 38,656 adults aged 18 years or older were included (Figure 1A). For the CHNS, a total of 7192 participants were excluded due to missing dietary data, anthropometric measurements, blood sample test results, or a prior diagnosis of hypertension, diabetes, and related cardiovascular diseases. Additionally, 30 pregnant or lactating women were excluded, along with 217 participants reporting implausible total energy intake (Men: <800 kcal/day or >6000 kcal/day; Women: <600 kcal/day or >4000 kcal/day). Furthermore, 6893 participants who participated only in one wave and 5090 participants who had dyslipidemia or took medication for dyslipidemia at baseline were excluded. Ultimately, a total of 3974 adults aged 18 years and above were selected as the study participants (Figure 1B).

The CFCS was approved by the Institutional Review Boards of the China National Center for Food Safety Risk Assessment (Protocol Number: No. 2025188). The CHNS was approved by the Institutional Review Boards of the University of North Carolina at Chapel Hill and the National Institute for Nutrition and Health, Chinese Center for Disease Control and Prevention (Protocol Number: No. 201524). All participants provided written informed consent for both the CFCS and the CHNS.

### 2.2. Dietary Assessment and Free Sugar Intake

Dietary data were collected using a non-consecutive three-day 24 h dietary recall method (where two adjacent 24 h dietary recall days were separated by at least three days) and a consecutive three-day 24 h dietary recall method for the CFCS and the CHNS, respectively. Three 24 h recalls were conducted for each participant, covering two weekdays and one weekend day.

Trained and certified interviewers visited households daily to conduct face-to-face interviews and collect information on all foods consumed by the participants. Dietary record forms were also used to assist the recall process, enhancing the completeness and accuracy of the dietary data. For food items and seasonings such as oils, salt, sugar and other condiments consumed by the participants during the survey period, interviewers employed a weighed inventory method. Food consumption at the household level was calculated based on the frequency of meals eaten at home and the ratio of the energy intake of all members.

During the dietary survey, the consumption of all food items for each participant was recorded using both the weighed dietary record method and the 24 h dietary recall method. Total energy and nutrients such as protein, fat, carbohydrates, and dietary sodium intake per day were calculated by matching individual food consumption records with the corresponding energy and nutrient composition data from the Chinese Food Composition Table [16,17].

A total of 1221 food samples were collected across multiple regions of China, covering all commonly consumed sugar-containing food categories: sugar-sweetened beverages, juices, baked products, sugar-sweetened dairy products, extruded puffed snacks, sugary snacks, candies and chocolates, and sugary sauces and condiments, as well as other sugary processed foods. Sampling was stratified by the production volume of each food category to ensure representativeness. High-performance Liquid Chromatography (HPLC) was used to determine the free sugars content of different kinds of food. For some foods, such as table sugars, honey and sauces, the free sugar content was obtained from the China Food Composition Table, with average values calculated for matched food entries. For foods containing intrinsic sugars (e.g., plain cow’s milk and yogurt), lactose and galactose were excluded from the final total free sugars calculation, in line with the WHO definition of free sugars. For a few food items with unavailable sugar content data, free sugar content was imputed using data from nutritionally comparable sugar-containing foods.

### 2.3. Definition of Dyslipidemia

Dyslipidemia was diagnosed and classified according to the “Guidelines for the Prevention and Treatment of Dyslipidemia in Chinese Adults (Revised Edition 2016)” [18]. The diagnosis involved four indicators: TC ≥ 6.2 mmol/L (hypercholesteremia); TG ≥ 2.3 mmol/L (hypertriglyceridemia); HDL-C < 1.0 mmol/L (hypo-HDL-cholesterolemia); and LDL-C ≥ 4.1 mmol/L (hyper-LDL-cholesterolemia). Dyslipidemia was defined as meeting any of the criteria.

### 2.4. Covariates and Indicators Measurement

Multiple covariates were included, such as sex, age, education level, place of residence, region of residence, income level, smoking history, alcohol consumption status in the past year, physical activity (PA), urbanization levels, body mass index (BMI), and intake of total energy, dietary protein, fat, carbohydrates, and sodium.

Age was categorized into three groups (18–44 years, 45–59 years, and ≥60 years). Educational level was categorized into three groups for the CFCS (primary school or below, secondary school, and college or above) and two groups for the CHNS (junior high school or below, and senior high school or above). Place of residence was separated into three groups for the CFCS (metropolises, small and medium-sized cities, and rural areas) and two groups for the CHNS (urban and rural). Region of residence was categorized into three groups for the CFCS (eastern, central, and western) and two groups for the CHNS (northern and southern) according to the geographical diversity of China. Per capita annual household income was categorized into five groups for the CFCS (CNY < 10,000, 10,000–39,999, 40,000–99,999, >100,000, and no response) and tertiles for the CHNS (low, medium, and high). Both smoking history and alcohol consumption status were categorized as yes or no (the CHNS only). All physical activity was self-reported by participants, including average weekly hours spent in leisure time, household chores, occupational activities, and transportation. Activity time was converted into metabolic equivalents of task (MET-hour/week) based on standards recommended by the American College of Sports Medicine and subsequently categorized into tertiles (low, medium, and high, the CHNS only) [19]. An urbanization index was calculated for each survey site based on the socioeconomics of the community, cultural, and developmental status, and urbanization levels were grouped into tertiles (low, medium, and high, the CHNS only). BMI was categorized into three groups (<18.5 kg/m^2^, 18.5–23.9 kg/m^2^, and ≥24.0 kg/m^2^, the CHNS only) which was calculated as body weight (kg) divided by the square of height (m^2^).

In the CHNS cohort, at least two trained nurses or health workers conducted anthropometric measurements on the participants with strict quality control. All measurement equipment was calibrated prior to use. Aminophenazone and cholesterol oxidase-phenol methods were used to measure concentrations of TC, TG, HDL-C and LDL-C. Height, weight and waist circumference were measured using a portable SECA206 stadiometer (seca, Hamburg, Germany), an electronic weight scale and an inelastic flexible ruler. Blood pressure measurements were performed using a standard mercury sphygmomanometer via the auscultation of Korotkoff sounds. All measurements were conducted with participants in a seated position in a quiet room, following a minimum five-minute rest period and confirmation of an empty bladder. The final blood pressure for each participant was determined by calculating the mean of three consecutive readings obtained under these standardized conditions. A Roche 702 automatic analyzer (Roche, Basel, Switzerland) was employed to measure fasting plasma glucose, utilizing the hexokinase enzymatic method.

### 2.5. Statistical Analysis

Categorical variables were described using frequencies and percentages and compared using the chi-square test. Continuous variables were presented as means and standard deviations and compared using the Kruskal–Wallis rank-sum test. In the CFCS, free sugar intake among participants was expressed as the mean (standard deviation [SD]), median (interquartile range: P25–P75), and P95. For the CHNS, the cut-off values for free sugar intake grouping were determined based on the population-level distribution of dietary free sugar. The 5 g/d threshold corresponds to the mean free sugar intake of the study population, and the 20 g/d threshold corresponds to the 95th percentile (P95) of the intake distribution. In accordance with the established principle of dietary exposure assessment, populations with intake above the P95 are conventionally classified as the high-exposure group, which informed our selection of these two cut-off ranges for analyses. Multivariable Cox proportional hazards regression models were used to examine the association between free sugar intake and the risk of dyslipidemia and its subtypes, with results expressed as hazard ratios (HRs) and 95% confidence intervals (CIs). A series of statistical models were constructed by sequential adjustment for different sets of covariates. Model 1 was adjusted for baseline age, sex, educational level, place of residence, region of residence, and per capita annual household income. Model 2 was further adjusted for alcohol consumption status, smoking history, physical activity level, and urbanization index based on Model 1. Model 3 included additional adjustments for total energy intake, and dietary intakes of protein, fat, carbohydrates, and sodium, as well as body mass index (BMI) based on Model 2. During the three rounds of follow-up, follow-up was discontinued for participants once they developed incident dyslipidemia. Follow-up duration was defined as the time interval from the baseline date to the date of outcome occurrence.

Stratified analyses were conducted based on key covariates, and interaction tests were performed to assess whether these stratification factors modify the association between free sugar intake and the risk of dyslipidemia. To investigate whether dietary free sugar intake exerts differential health effects across intake levels and for distinct subtypes of dyslipidemia, as well as the potential non-linear dose–response relationship between dietary free sugar intake and the hazard ratios (HRs) of dyslipidemia, we conducted multivariable-adjusted restricted cubic spline (RCS) regression analyses [20]. All statistical analyses were performed using SAS software (version 9.4; SAS Institute Inc., Cary, NC, USA), and figures were generated with R software (version 4.4.1). A two-tailed *p*-value < 0.05 was considered to indicate statistical significance.

## 3. Results

### 3.1. Population Characteristics of Study Participants in the CFCS and the CHNS

Table 1 presents the basic characteristics of participants and free sugar intake in the CFCS. A total of 38,656 participants were included in the CFCS, with a mean free sugar intake of 6.4 g/d. Significant differences in free sugar intake were observed across sociodemographic subgroups. Specifically, higher free sugar intake was observed among participants aged 18–44 years (7.9 g/d), those with a college education or above (9.8 g/d), those living in metropolitan areas (8.2 g/d) or eastern China (7.6 g/d), and those with a per capita annual household income above CNY 100,000 (9.7 g/d). In contrast, lower free sugar intake was found among those aged 60 years and above (5.2 g/d), those with a primary school education or below (4.4 g/d), those living in rural areas (5.3 g/d) or western China (5.5 g/d), and those with a per capita annual household income below CNY 10,000 (4.9 g/d).

Table 2 summarizes the sociodemographic and baseline characteristics of the participants, stratified by free sugar intake levels in the CHNS cohort. Participants were classified into three groups according to their intake of free sugar: <5 g/d (*n* = 2721), 5–20 g/d (*n* = 1052), and >20 g/d (*n* = 201). Compared with participants in the reference group (free sugar intake < 5 g/d), those with higher free sugar intake were more likely to have higher educational levels, reside in urban areas, have lower physical activity levels, have a higher annual individual income, and live in areas with higher urbanization levels, as well as higher intakes of energy, protein, fat, and carbohydrates (all *p* < 0.05).

**Figure 2:**
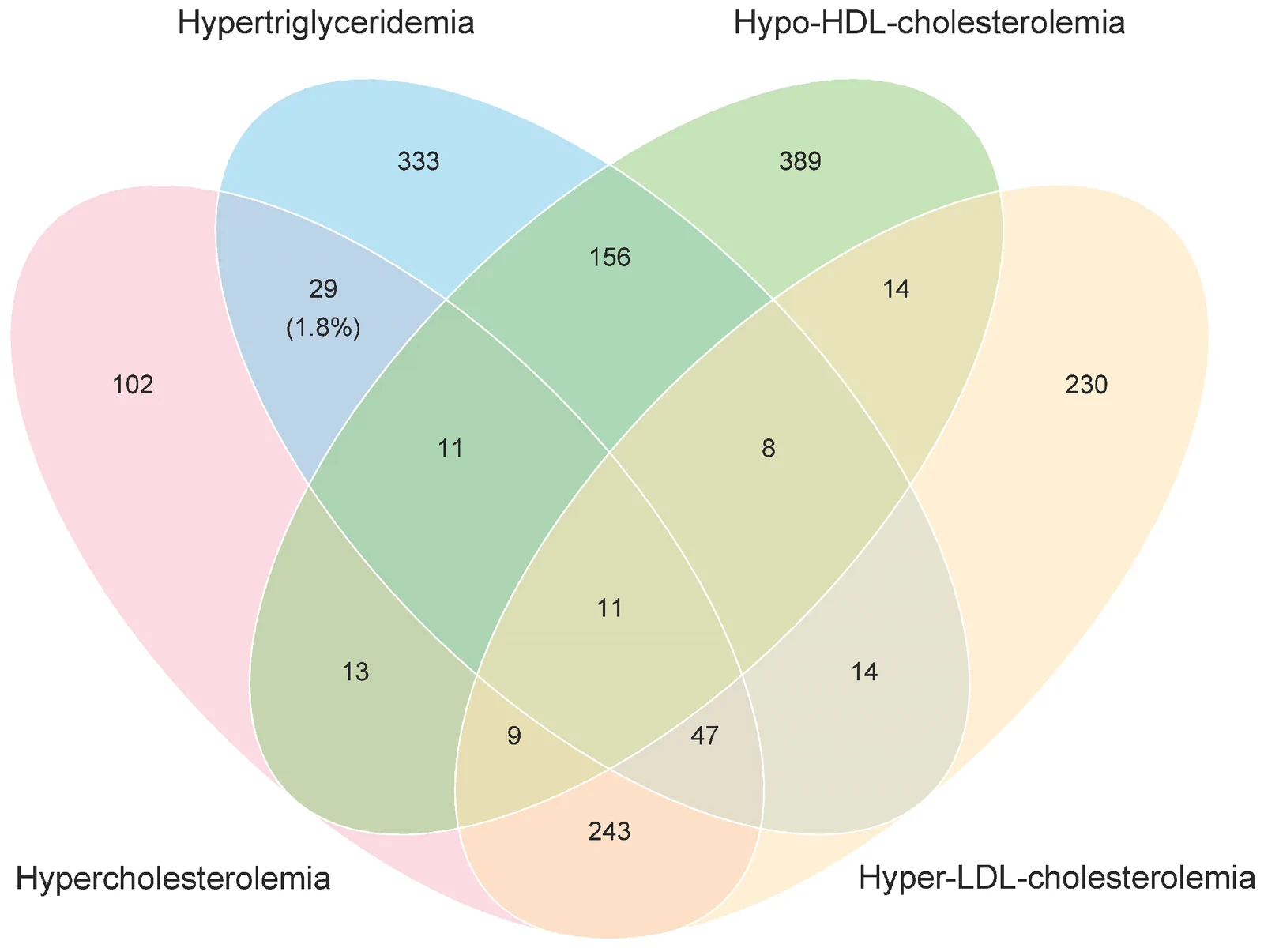
Venn diagram of sample distribution for different subtypes of dyslipidemia.

A total of 3974 participants were included in the CHNS. During follow-up, 1052 participants developed incident dyslipidemia, corresponding to a cumulative incidence of 26.5%. Among participants with dyslipidemia, 465 had hypercholesterolemia, 609 had hypertriglyceridemia, 611 had hypo-HDL-cholesterolemia, and 576 had hyper-LDL-cholesterolemia. A proportion of participants had two or more concurrent dyslipidemia subtypes; 11 participants presented with all four subtypes. The distribution and combined patterns of these dyslipidemia subtypes are presented in Figure 2.

### 3.2. Association of Free Sugar Intake with Dyslipidemia and Its Subtypes

Table 3 summarizes the associations between free sugar intake levels and dyslipidemia and its subtypes in the CHNS cohort. The cohort accrued a total of 24,531 person-years of follow-up, with a median follow-up of 6.0 years (IQR: 3.0–9.0 years). After adjustment for a range of potential confounders (sex, age, education level, residence type, geographic region, per capita annual household income, smoking history, alcohol consumption status, physical activity level [measured in metabolic equivalents], urbanization index, BMI, and dietary intakes of energy, protein, fat, carbohydrates, and sodium), participants with a free sugar intake > 20 g/d had a 38% higher risk of dyslipidemia (HR = 1.38, 95% CI: 1.04–1.84; *p* for trend = 0.006), compared with the reference group (<5 g/d free sugar intake).

For individual dyslipidemia subtypes, after multivariable adjustment, compared with the reference group (<5 g/d free sugar intake), participants with an intake > 20 g/d had a 91% higher risk of hypo-HDL-cholesterolemia (HR = 1.91, 95% CI: 1.34–2.74; *p* for trend < 0.001) and a 52% higher risk of hyper-LDL-cholesterolemia (HR = 1.52, 95% CI: 1.01–2.29; *p* for trend = 0.004), respectively. In contrast, no significant associations were observed between free sugar intake and the risks of hypercholesterolemia or hypertriglyceridemia (all *p* > 0.05).

We further conducted sensitivity analyses to validate the robustness of the observed association. We calculated the proportion of total daily energy contributed by free sugars for each participant and categorized participants into quintiles based on this energy-adjusted metric. Compared with participants in the lowest quintile of free sugar intake (% of total energy), those in the highest quintile had a 26% elevated risk of dyslipidemia (HR = 1.26, 95% CI: 1.05–1.52).

### 3.3. Stratified Analyses of Free Sugar Intake with Risk of Dyslipidemia

Table 4 summarizes the results of stratified analyses and interaction tests examining the associations between free sugar intake levels and dyslipidemia risk. The positive association between free sugar intake and dyslipidemia risk was statistically significant in women, adults aged 18–44 years, participants with higher educational levels, urban residents, and those living in northern China (all *p* < 0.05). Notably, the residential region significantly modified the association between free sugar intake and dyslipidemia risk (*p* for interaction < 0.05).

The results of stratified and interaction analyses for individual dyslipidemia subtypes are provided in Supplementary Materials: Tables S1–S4. Briefly, the positive association between free sugar intake and hypo-HDL-cholesterolemia risk was statistically significant across multiple subgroups: women, adults aged 18–44 years, rural residents, those living in northern China, participants with moderate metabolic equivalent (MET) levels, those living in areas with low urbanization levels, and those with BMI ≥ 24.0 kg/m^2^ (all *p* < 0.01). For hyper-LDL-cholesterolemia, the positive association was significant in participants with low education levels, urban residents, those in southern China, participants with higher annual individual income, and those living in areas with high urbanization levels (all *p* < 0.05).

### 3.4. Nonlinear Associations Between Free Sugar Intake and the Risk of Dyslipidemia and Its Subtypes

**Figure 3:**
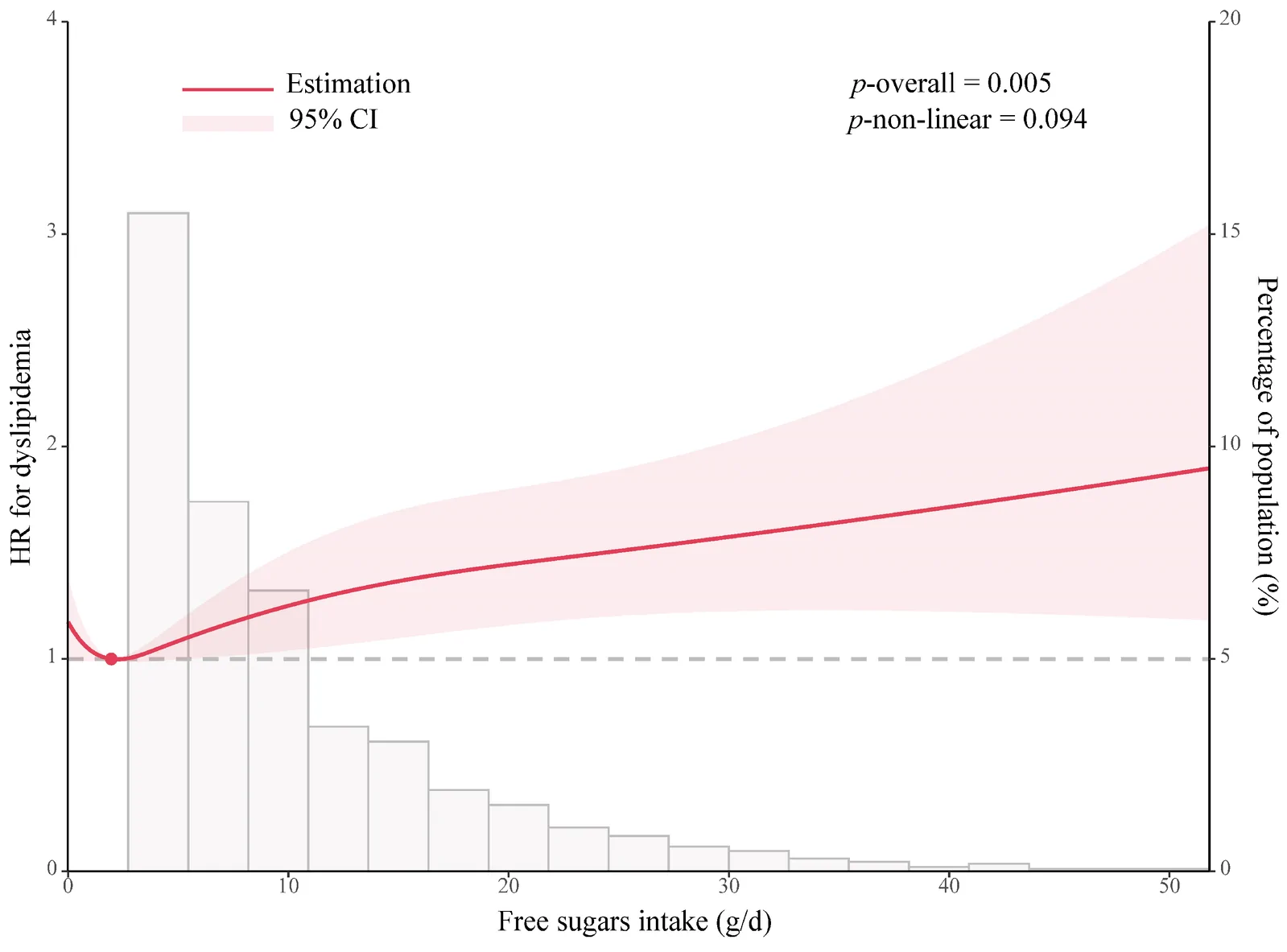
The dose–response relationship between free sugar intake and dyslipidemia.

We performed dose–response analyses to examine the association between free sugar intake and the risk of dyslipidemia and its subtypes. As shown in Figure 3, there was a linear dose–response association between free sugar intake and overall dyslipidemia risk (*p* for nonlinearity = 0.094), indicating that the hazard ratio for dyslipidemia increased progressively with higher free sugar intake.

**Figure 4:**
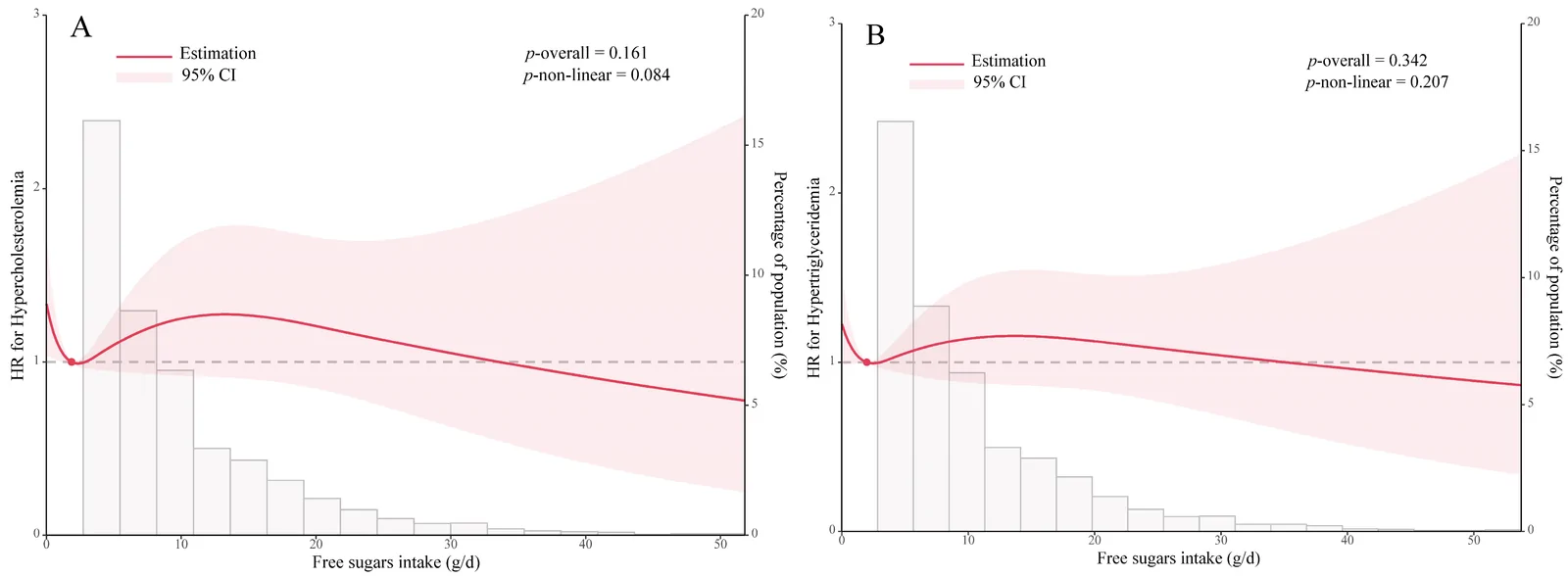

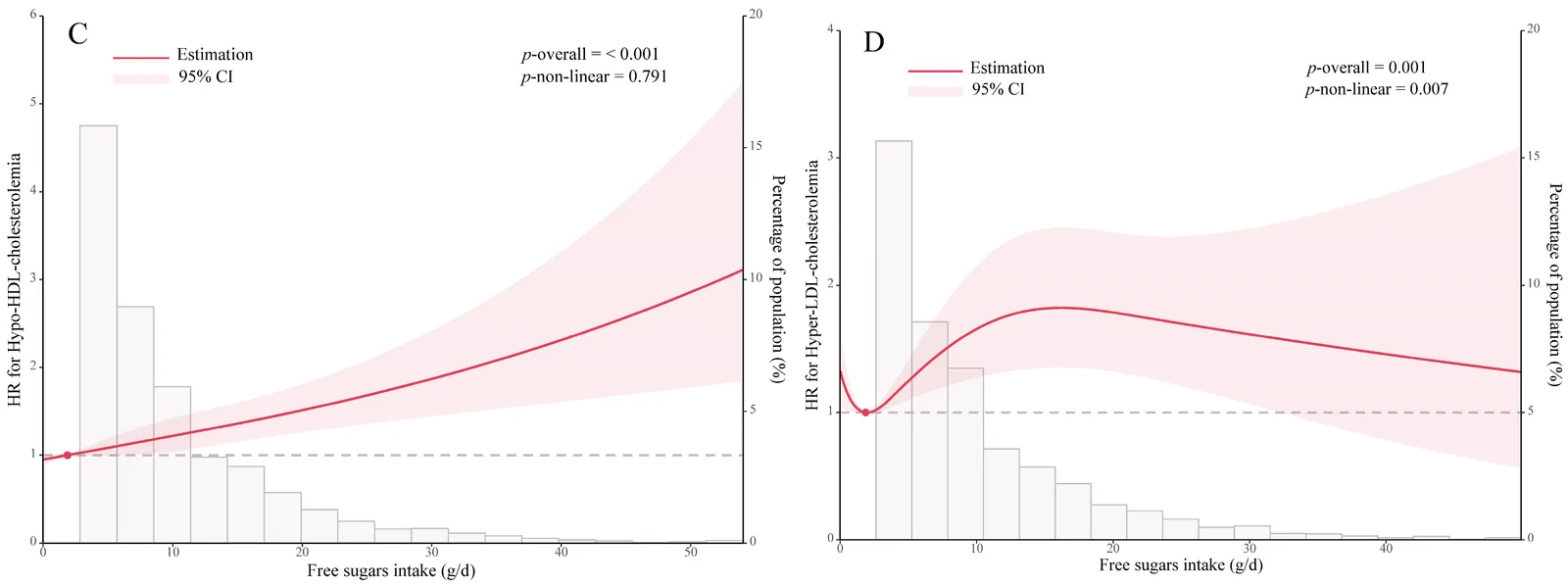
The dose–response relationship between free sugar intake and different subtypes of dyslipidemia. (**A**) RCS regression models for free sugar intake and HRs of hypercholesterolemia. (**B**) RCS regression models for free sugar intake and HRs of hypertriglyceridemia. (**C**) RCS regression models for free sugar intake and HRs of hypo-HDL-cholesterolemia. (**D**) RCS regression models for free sugar intake and HRs of hyper-LDL-cholesterolemia.

A similar linear association was observed for hypo-HDL-cholesterolemia risk (Figure 4C). For hyper-LDL-cholesterolemia, however, the association followed a significant nonlinear inverted U-shaped curve (*p* for nonlinearity = 0.007; Figure 4D): the risk first increased with the intake level and then declined after the inflection point, but the overall linear trend was not statistically significant. No significant dose–response relationships were detected for hypercholesterolemia or hypertriglyceridemia.

## 4. Discussion

Overall, using data from the CFCS and the CHNS, we systematically characterized free sugar intake levels among Chinese adults and evaluated their associations with the risk of incident dyslipidemia and its subtypes. For the CHNS, our findings demonstrated that higher free sugar intake was independently associated with increased dyslipidemia risk in a linear dose–response pattern. Specifically, a free sugar intake above 20 g/d was associated with significantly elevated risks of incident hypo-HDL-cholesterolemia and hyper-LDL-cholesterolemia. Notably, we observed an inverted U-shaped nonlinear association between free sugar intake and hyper-LDL-cholesterolemia risk.

Over recent decades, population-level free sugar intake has risen substantially, and extensive research has examined its associations with chronic noncommunicable diseases (NCDs), including overweight/obesity [8,21], MetS [22], cardiovascular disease [7,23], and dyslipidemia [24,25]. Overall, findings from prior studies are largely consistent with the results of our study.

Most consistently, multiple lines of evidence support a robust association between a higher intake of added sugars (largely equivalent to free sugars) and reduced HDL-C levels, as well as an increased risk of hypo-HDL-cholesterolemia. In a cross-sectional analysis of the 1999–2004 US NHANES, Welsh et al. identified an inverse linear trend between added sugars intake and HDL-C levels among adolescents (*p* for linear trend = 0.007), though no significant trends were detected for LDL-C (*p* for trend = 0.10) or TC (*p* for trend = 0.29) [26]. A systematic review further corroborated this association, reporting that added sugars intake from sugar-sweetened beverages (SSBs) and coffee drinks was linked to a higher prevalence of hypo-HDL-cholesterolemia [27]. Notably, a population-based prospective cohort of Hispanic/Latino adults in the US demonstrated a significant dose–response relationship: participants in the highest quintile of added sugars intake had HDL-C levels 2.5 mg/dL lower than those in the lowest quintile (95% CI: −4.08 to −0.96), and consuming > 2 servings of sugar-sweetened beverages daily was associated with a 35% higher risk of incident hypo-HDL-cholesterolemia (incidence rate ratio = 1.35, 95% CI: 1.02–1.79) [28]. For LDL-C and triglycerides, evidence has been more heterogeneous. In a repeated-measures experimental study among adult males, Le et al. found that a high-fructose diet (1.5 g/kg body weight per day) led to elevated TG and LDL-C levels [29].

Several potential biological mechanisms may underlie the observed association between dietary free sugar intake and dyslipidemia risk. Chronic high intake of free sugars (including sucrose and high-fructose corn syrup, predominantly consumed via sugar-sweetened beverages) is a well-established modifiable risk factor for atherogenic dyslipidemia, with fructose serving as the key pathogenic driver [30]. Fructose is primarily metabolized in the liver by fructokinase C, an enzyme lacking negative feedback regulation by cellular energy status. This property allows fructose to bypass key glycolytic checkpoints, thereby robustly inducing de novo lipogenesis (DNL), driving triglyceride synthesis, and promoting the excessive secretion of very low-density lipoprotein (VLDL) [31]. Meanwhile, since fructose triggers only minimal insulin release, hepatic production of apolipoprotein CIII (apoCIII) remains unrepressed. Elevated apoCIII exacerbates hypertriglyceridemia by inhibiting lipoprotein lipase activity and impairing the clearance of triglyceride-rich lipoproteins (TRLs) [32]. Accumulated TRLs then undergo remodeling mediated by cholesterol ester transfer protein (CETP), generating small dense LDL (sdLDL) particles and dysfunctional HDL [31]. Beyond direct effects on hepatic lipid metabolism, fructose further promotes visceral adiposity and hepatic insulin resistance via the diacylglycerol/novel protein kinase C axis, creating a self-reinforcing vicious cycle that perpetuates lipid dysregulation [31]. Additional factors including hyperuricemia, gut dysbiosis, and chronic low-grade inflammation act synergistically to amplify these metabolic perturbations [30,33].

Global public health authorities have increasingly prioritized dietary sugar restriction as a core strategy for chronic disease prevention. In 2015, the World Health Organization (WHO) released global guidelines recommending lifelong restriction of free sugar intake, strongly advising a limit of less than 10% of total daily energy intake, with a conditional recommendation for further reduction to below 5% for additional health benefits [6]. In China, the Chinese Dietary Guidelines 2022 recommend limiting added sugar intake to no more than 50 g/d, with an optimal target of less than 25 g/d [34]. Notably, our findings indicate that free sugar intake among Chinese adults falls below the recommended limit set out in these guidelines. The newly released 2025–2030 Dietary Guidelines for Americans provide a strict, evidence-based stance against added and non-nutritive sweeteners, explicitly stating that no quantity of these sweeteners is recommended as part of a healthy diet. In practical terms, the updated guidelines set a stringent limit of ≤10 g of added sugars per meal, replacing the previous population-level threshold of less than 10% of total daily energy. Notably, the guidelines extend the recommended age for avoiding added sugars from less than 2 years to less than 10 years [35]. Consistent with these policy recommendations, mounting epidemiological evidence has linked free sugar intake—particularly in the form of SSBs—to an elevated risk of multiple adverse health outcomes, including weight gain [36,37], type 2 diabetes [38], MetS [39,40], and other NCDs [41,42]. An umbrella review of published meta-analyses further demonstrated that high versus low SSBs consumption was significantly associated with reduced HDL-C (weighted mean difference: −1.46, 95% CI: −2.25 to −0.67) and elevated LDL-C (weighted mean difference: 1.21, 95% CI: 0.23 to 2.20) [43], which aligns with the dyslipidemia-related findings observed in our study. Nevertheless, China is currently undergoing rapid economic development and lifestyle transitions, which have driven substantial shifts in dietary behaviors and patterns. Traditional Chinese dietary patterns are increasingly shifting toward Western-style diets, accompanied by rising consumption of packaged foods and beverages, particularly SSBs [44]. A cross-sectional study among Chinese children and adolescents has already found that participants with the highest SSBs intake (≥201.7 mL/day) had a 0.10 mmol/L (95% CI: 0.02–0.18) higher TC and 0.09 mmol/L (95% CI: 0.03–0.16) higher LDL-C concentration compared with non-consumers (0 mL/day) [45]. Given the continuously rising prevalence of chronic NCDs including dyslipidemia in the Chinese population, future efforts should continue to monitor temporal trends in population-level free sugar intake.

Our findings demonstrate that higher free sugar intake is associated with an increased risk of dyslipidemia in specific subgroups: women, adults aged 18–44 years, individuals with a senior high school education or above, urban residents, residents of northern China, people with higher per capita annual household income, and those with low physical activity levels. Stratified analyses by dyslipidemia subtypes further showed that higher free sugar intake was linked to elevated risks of hypo-HDL-cholesterolemia and hyper-LDL-cholesterolemia among participants with higher per capita annual household income and those with moderate or low physical activity. Notably, no significant association was observed between free sugar intake and incident hypertriglyceridemia, consistent with findings from large prospective cohorts. The Hispanic Community Health Study/Study of Latinos reported no linear link between the highest quintile of added sugars intake and elevated triglycerides after full confounder adjustment, while the inverse association with HDL-C remained robust [28]. Two factors may account for this subtype-specific inconsistency. First, the triglyceride-elevating effect of free sugar has a clear dose threshold. The 2022 EFSA report confirmed consistent triglyceride elevation only at high fructose intakes (>100 g/day, >20% of total energy), with inconsistent evidence at lower levels [46]. The relatively low free sugar intake among adults in the CHNS may fall below this threshold. Second, solid-dominant sugar sources in Chinese diets also exert weaker metabolic effects than liquid sugars prevalent in Western diets [47].

These subgroup disparities are closely tied to ongoing dietary and lifestyle transitions in China. With rapid urbanization and rising household incomes, dietary patterns have shifted substantially away from traditional diets toward Westernized patterns [44]. Traditional Chinese diets are characterized by high consumption of cereals and vegetables and low consumption of animal-source foods [48]. As household income rises and processed foods, snacks, desserts, and SSBs become more accessible, these items have become increasingly prevalent in daily diets. As major dietary sources of free sugar, their growing consumption has driven a corresponding rise in population-level free sugar intake [49,50]. Furthermore, lifestyle changes including reduced physical activity are also well-established risk factors for dyslipidemia [2,51,52]. The co-occurrence of these multiple adverse factors therefore explains the substantially higher dyslipidemia risk observed in high-risk subgroups. Moreover, moderate free sugar intake (5–20 g/d) was associated with an elevated dyslipidemia risk in our cohort, yet this vulnerable subgroup remains largely overlooked in conventional interventions focused exclusively on high sugar consumers. Accordingly, enhanced health education and dietary guidance should also be extended to this population to adopt a balanced dietary lifestyle, thereby reducing the burden of dyslipidemia and other chronic diseases.

To the best of our knowledge, this is the first study to use data from the CFCS and the CHNS to examine free sugar intake levels and their associations with dyslipidemia and its subtypes among Chinese adults. In addition, we applied a multivariable Cox proportional hazards model to investigate the longitudinal associations between free sugar intake and the risk of dyslipidemia. Nevertheless, several limitations of this study should be acknowledged. First, dietary data collected using the 24 h recall method may be susceptible to recall bias. Second, although we adjusted for a set of major confounding factors, residual confounding cannot be entirely excluded. Third, dietary intake estimated from 24 h recalls may not fully reflect long-term dietary habits. Future studies incorporating food frequency questionnaire (FFQ) data are warranted to further examine the associations between sugar-related dietary behaviors and metabolic health outcomes.

## 5. Conclusions

In conclusion, our findings indicate that free sugar intake levels among Chinese adults are relatively low compared to national dietary recommendations. Higher free sugar intake is independently and positively associated with an increased risk of incident dyslipidemia, as well as its subtypes, hypo-HDL-cholesterolemia and hyper-LDL-cholesterolemia. Urban residents and individuals with low physical activity levels are more susceptible to hypo-HDL-cholesterolemia and hyper-LDL-cholesterolemia in the context of high dietary free sugar intake. Given the rising trend of free sugar intake and the growing prevalence of dyslipidemia in the general population, targeted nutrition interventions focusing on high-risk subgroups are warranted to promote healthy balanced diets, improve overall dietary quality, and reduce the population-level burden of dyslipidemia.

## Figures and Tables

**Table 1:** Basic characteristics of overall participants and free sugar intake in the CFCS.

	*n* (%)	Intake of Free Sugars (g/d)						*p*-Value
		Mean	SD	Median	P25	P75	P95	
Sex								<0.001
Men	18,566 (48.0)	6.4	13.6	1.1	0.0	8.1	27.4	
Women	20,090 (52.0)	6.5	11.6	1.7	0.0	8.8	26.3	
Age								<0.001
18–44	16,060 (41.5)	7.9	15.2	2.7	0.0	10.7	30.8	
45–59	14,907 (38.6)	5.4	10.3	0.7	0.0	7.1	24.0	
≥60	7689 (19.9)	5.2	9.9	0.8	0.0	7.2	22.8	
Education level								<0.001
Primary school or below	8392 (21.7)	4.4	10.5	0.2	0.0	5.3	20.6	
Secondary school	21,117 (54.6)	5.8	10.5	1.0	0.0	7.6	25.0	
College or above	9147 (23.7)	9.8	17.2	4.7	0.1	13.7	34.5	
Place of residence								<0.001
Metropolis	12,094 (31.3)	8.2	15.2	3.2	0.0	11.2	30.3	
Small and medium-sized cities	12,478 (32.3)	6.0	12.2	1.2	0.0	8.0	26.2	
Rural areas	14,084 (36.4)	5.3	10.0	0.5	0.0	6.7	23.9	
Region of residence								<0.001
Eastern regions	18,258 (47.2)	7.6	14.1	2.7	0.0	10.3	29.2	
Central regions	11,246 (29.1)	5.3	10.8	0.6	0.0	6.9	24.0	
Western regions	9152 (23.7)	5.5	11.1	0.7	0.0	7.1	24.3	
Individual annual income								<0.001
<10,000	9552 (24.7)	4.9	10.5	0.3	0.0	6.0	23.0	
10,000–39,999	21,088 (54.6)	6.1	12.0	1.1	0.0	7.9	25.7	
40,000–99,999	4843 (12.5)	9.6	15.9	4.5	0.1	13.3	34.7	
>100,000	1192 (3.1)	9.7	16.1	4.6	0.1	14.1	33.6	
No response	1981 (5.1)	7.9	14.6	3.2	0.0	11.0	29.0	
Total	38,656 (100.0)	6.4	12.6	1.4	0.0	8.6	26.9	

**Table 2:** Basic characteristics of overall participants according to different levels of free sugar intake in the CHNS.

	Intake of Free Sugars (g/d)			*p*-Value
	<5	5–20	>20	
Sex				0.293
Men	1188 (43.7)	450 (42.8)	98 (48.8)	
Women	1533 (56.3)	602 (57.2)	103 (51.2)	
Age				0.039
18–44	1051 (38.6)	427 (40.6)	91 (45.3)	
45–59	1093 (40.2)	378 (35.9)	65 (32.3)	
≥60	577 (21.2)	247 (23.5)	45 (22.4)	
Education level				<0.001
Junior high school or below	2162 (79.5)	641 (60.9)	85 (42.3)	
Senior high school or above	559 (20.5)	411 (39.1)	116 (57.7)	
Place of residence				<0.001
Urban areas	673 (24.7)	460 (43.7)	133 (66.2)	
Rural areas	2048 (75.3)	592 (56.3)	68 (33.8)	
Region of residence				0.020
Northern regions	1013 (37.2)	433 (41.2)	89 (44.3)	
Southern regions	1708 (62.8)	619 (58.8)	112 (55.7)	
Individual annual income				<0.001
Low	1113 (40.9)	263 (25.0)	35 (17.4)	
Medium	971 (35.7)	326 (31.0)	41 (20.4)	
High	637 (23.4)	463 (44.0)	125 (62.2)	
Smoking history				0.003
Yes	747 (27.5)	239 (22.7)	42 (20.9)	
No	1974 (72.5)	813 (77.3)	159 (79.1)	
Drinking past year				0.323
Yes	806 (29.6)	287 (27.3)	55 (27.4)	
No	1915 (70.4)	765 (72.7)	146 (72.6)	
Physical activity				<0.001
Low	760 (27.9)	360 (34.2)	63 (31.3)	
Medium	881 (32.4)	419 (39.8)	90 (44.8)	
High	1080 (39.7)	273 (26.0)	48 (23.9)	
Urbanization				<0.001
Low	1170 (43.0)	207 (19.7)	31 (15.4)	
Medium	861 (31.6)	399 (37.9)	53 (25.4)	
High	690 (25.4)	446 (42.4)	117 (58.2)	
BMI (kg/m^2^)				0.570
<18.5	202 (7.4)	62 (5.9)	13 (6.5)	
18.5–23.9	1611 (59.2)	628 (59.7)	121 (60.2)	
≥24.0	908 (33.4)	362 (34.4)	67 (33.3)	
Energy (kcal/d)	2199.2 ± 702.7	2188.1 ± 690.2	2408.1 ± 748.0	<0.001
Protein (g/d)	68.7 ± 24.9	75.2 ± 30.2	82.8 ± 31.4	<0.001
Fat (g/d)	79.4 ± 42.0	83.4 ± 37.2	91.8 ± 43.3	<0.001
Carbohydrates (g/d)	292.4 ± 112.4	273.2 ± 108.6	302.7 ± 113.7	<0.001
Sodium (mg/d)	4888.6 ± 4290.0	5273.7 ± 4518.7	6293.3 ± 8658.1	0.003
WC (cm)	80.30 ± 10.03	80.94 ± 10.35	80.20 ± 10.17	0.243
SBP (mmHg)	120.10 ± 15.26	120.37 ± 15.15	119.82 ± 14.81	0.847
DBP (mmHg)	78.00 ± 9.94	78.16 ± 9.00	78.02 ± 8.82	0.861
FPG (mmol/L)	5.09 ± 0.84	5.09 ± 0.88	5.04 ± 0.78	0.565

For categorical variables, values are reported as frequencies with corresponding percentages; for continuous variables, results are presented as means ± SD.

**Table 3:** Associations of dietary free sugar intake with risk of dyslipidemia and its subtypes.

	Intake of Free Sugars (g/d)			*p*-Trend
	<5	5–20	>20	
Dyslipidemia ^a^				
Model 1	1.00 (ref)	1.16 (1.01, 1.33) *	1.29 (0.98, 1.33)	0.017
Model 2	1.00 (ref)	1.15 (0.99, 1.32)	1.29 (0.97, 1.71)	0.023
Model 3	1.00 (ref)	1.16 (1.01, 1.34) *	1.38 (1.04, 1.84) *	0.006
Hypercholesteremia ^b^				
Model 1	1.00 (ref)	1.02 (0.82, 1.27)	0.72 (0.40, 1.30)	0.470
Model 2	1.00 (ref)	1.05 (0.84, 1.30)	0.74 (0.41, 1.34)	0.593
Model 3	1.00 (ref)	1.05 (0.84, 1.31)	0.77 (0.43, 1.39)	0.688
Hypertriglyceridemia ^c^				
Model 1	1.00 (ref)	1.02 (0.83, 1.21)	0.88 (0.57, 1.21)	0.650
Model 2	1.00 (ref)	0.99 (0.82, 1.21)	0.89 (0.57, 1.37)	0.662
Model 3	1.00 (ref)	0.99 (0.83, 1.21)	0.97 (0.62, 1.51)	0.919
Hypo-HDL-Cholesterolemia ^d^				
Model 1	1.00 (ref)	1.29 (1.07, 1.55) **	1.78 (1.25, 2.52) **	<0.001
Model 2	1.00 (ref)	1.27 (1.06, 1.53) *	1.78 (1.25, 2.53) **	<0.001
Model 3	1.00 (ref)	1.28 (1.06, 1.54) **	1.91 (1.34, 2.73) **	<0.001
Hyper-LDL-Cholesterolemia ^e^				
Model 1	1.00 (ref)	1.29 (1.07, 1.55) **	1.42 (0.95, 2.13)	0.007
Model 2	1.00 (ref)	1.27 (1.05, 1.53) *	1.40 (0.93, 2.11)	0.012
Model 3	1.00 (ref)	1.28 (1.06, 1.55) *	1.52 (1.01, 2.29) *	0.004

^a^ *n* = 3974. ^b^ *n* = 5310. ^c^ *n* = 4934. ^d^ *n* = 5069. ^e^ *n* = 5187. * *p* < 0.05, ** *p* < 0.01. Model 1 was adjusted for sex, age, education level, residence type, geographic region, per capita annual household income. Model 2 was further adjusted for smoking history, alcohol consumption status, metabolic equivalents, and urbanization index based on Model 1. Model 3 was further adjusted for BMI, intakes of energy, protein, fat, carbohydrates, and sodium based on Model 2. When examining the association between free sugar intake and dyslipidemia, we excluded participants with prevalent dyslipidemia at baseline. For analyses of individual dyslipidemia subtypes, we excluded only participants who had the corresponding prevalent subtype at baseline.

**Table 4:** Stratified analyses of free sugar intake and dyslipidemia.

	Intake of Free Sugars (g/d)			*p* for Interaction
	<5	5–20	>20	
Sex				0.220
Men	1.00 (ref)	1.08 (0.87, 1.34)	1.25 (0.83, 1.88)	
Women	1.00 (ref)	1.25 (1.03, 1.52) *	1.51 (1.01, 2.25) *	
Age				0.127
18–44	1.00 (ref)	1.29 (1.03, 1.61) *	1.85 (1.23, 2.78) **	
45–59	1.00 (ref)	0.98 (0.79, 1.23)	1.17 (0.73, 1.86)	
≥60	1.00 (ref)	1.39 (0.99, 1.94)	1.11 (0.51, 2.39)	
Education level				0.077
Junior high school or below	1.00 (ref)	1.06 (0.89, 1.27)	1.35 (0.89, 2.03)	
Senior high school or above	1.00 (ref)	1.39 (1.08, 1.79) *	1.55 (1.02, 2.34) *	
Place of residence				0.145
Urban areas	1.00 (ref)	1.37 (1.08, 1.74) *	1.56 (1.07, 2.27) *	
Rural areas	1.00 (ref)	1.05 (0.88, 1.26)	1.33 (0.83, 2.11)	
Region of residence				0.044
Northern regions	1.00 (ref)	1.29 (1.03, 1.61) *	1.77 (1.18, 2.66) **	
Southern regions	1.00 (ref)	1.12 (0.93, 1.35)	1.20 (0.80, 1.79)	
Individual annual income				0.061
Low	1.00 (ref)	1.04 (0.80, 1.35)	1.70 (0.99, 2.91)	
Medium	1.00 (ref)	1.04 (0.80, 1.35)	1.15 (0.63, 2.09)	
High	1.00 (ref)	1.37 (1.08, 1.74) *	1.28 (0.84, 1.95)	
Smoking history				0.745
Yes	1.00 (ref)	0.91 (0.69, 1.21)	1.57 (0.96, 2.58)	
No	1.00 (ref)	1.26 (1.06, 1.49) **	1.26 (0.89, 1.79)	
Drinking past year				0.185
Yes	1.00 (ref)	1.08 (0.84, 1.40)	1.02 (0.59, 1.76)	
No	1.00 (ref)	1.19 (0.99, 1.41)	1.66 (1.18, 2.32) **	
Physical activity				0.653
Low	1.00 (ref)	1.31 (1.01, 1.71) *	1.14 (0.63, 2.05)	
Medium	1.00 (ref)	1.25 (0.99, 1.57)	1.48 (0.97, 2.26)	
High	1.00 (ref)	0.98 (0.75, 1.27)	1.49 (0.88, 2.52)	
Urbanization				0.695
Low	1.00 (ref)	1.05 (0.77, 1.42)	1.46 (0.77, 2.78)	
Medium	1.00 (ref)	1.17 (0.92, 1.49)	1.49 (0.84, 2.65)	
High	1.00 (ref)	1.18 (0.94, 1.49)	1.27 (0.86, 1.87)	
BMI (kg/m^2^)				0.327
<18.5	1.00 (ref)	1.23 (0.64, 2.40)	1.72 (0.58, 5.13)	
18.5–23.9	1.00 (ref)	1.19 (0.99, 1.45)	1.53 (1.08, 2.16) *	
≥24.0	1.00 (ref)	1.08 (0.86, 1.37)	0.93 (0.53, 1.64)	

* *p* < 0.05, ** *p* < 0.01.

## Data Availability

The original contributions presented in this study are included in the article/supplementary material. Further inquiries can be directed to the corresponding authors.

## Supplementary Materials

The following supporting information can be downloaded at https://www.mdpi.com/article/10.3390/foods15162945/s1, Table S1: Stratified analyses of free sugar intake and hypercholesteremia; Table S2: Stratified analyses of free sugar intake and hypertriglyceridemia; Table S3: Stratified analyses of free sugar intake and hypo-HDL-cholesterolemia; Table S4: Stratified analyses of free sugar intake and hyper-LDL-cholesterolemia.

## Abbreviations

The following abbreviations are used in this manuscript:

WHO	World Health Organization
CFCS	Chinese Food Consumption Survey
CHNS	China Health and Nutrition Survey
NHANES	National Health and Nutrition Examination Survey
TC	Total cholesterol
TG	Triglyceride
HDL-C	High-density lipoprotein cholesterol
LDL-C	Low-density lipoprotein cholesterol
BMI	Body mass index
SSBs	Sugar-sweetened beverages
MetS	Metabolic syndrome
NCDs	Noncommunicable diseases
RCS	Restricted cubic spline
HR	Hazard ratio
CI	Confidence interval
DNL	De novo lipogenesis
VLDL	Very low-density lipoprotein
TRL	Triglyceride-rich lipoproteins
CETP	Cholesterol ester transfer protein
