# Supplementary material for: Association Between Free Sugar Intake and Risk of Dyslipidemia in Chinese Adults—Evidence from the Chinese Food Consumption Survey and the China Health and Nutrition Survey
Source: Foods. 2026 Aug 21;15(16):2945. doi: 10.3390/foods15162945 (PMC13512140; doi:10.3390/foods15162945)
Supplement: Supplementary file 1 [file foods-15-02945-s001.zip › foods-4483035-supplementary.pdf]

Table S1. Stratified analyses of free sugars intake with hypercholesteremia

	Intake of free sugars (g/d)			*P* for Interaction
	< 5	5–20	> 20	
Sex				0.240
Men	1.00 (ref)	0.86 (0.60, 1.22)	0.68 (0.29, 1.57)	
Women	1.00 (ref)	1.18 (0.89, 1.56)	0.79 (0.35, 1.80)	
Age				0.356
18–44	1.00 (ref)	1.23 (0.84, 1.79)	0.46 (0.11, 1.89)	
45–59	1.00 (ref)	0.94 (0.68, 1.30)	1.02 (0.47, 2.20)	
≥ 60	1.00 (ref)	1.05 (0.63, 1.73)	1.08 (0.32, 3.65)	
Education level				0.954
Junior high school or below	1.00 (ref)	1.00 (0.76, 1.31)	1.20 (0.59, 2.45)	
Senior high school or above	1.00 (ref)	1.16 (0.79, 1.69)	0.47 (0.17, 1.31)	
Place of residence				0.367
Urban areas	1.00 (ref)	1.13 (0.79, 1.61)	0.94 (0.46, 1.92)	
Rural areas	1.00 (ref)	1.01 (0.76, 1.34)	0.54 (0.17, 1.69)	
Region of residence				0.037
Northern regions	1.00 (ref)	1.32 (0.96, 1.83)	0.88 (0.38, 2.03)	
Southern regions	1.00 (ref)	0.89 (0.66, 1.21)	0.75 (0.33, 1.72)	
Individual annual income				0.341
Low	1.00 (ref)	1.07 (0.70, 1.62)	1.01 (0.31, 3.25)	
Medium	1.00 (ref)	0.85 (0.57, 1.27)	0.41 (0.10, 1.68)	
High	1.00 (ref)	1.22 (0.86, 1.75)	0.88 (0.40, 1.96)	
Smoking history				0.937
Yes	1.00 (ref)	1.02 (0.66, 1.56)	0.45 (0.11, 1.85)	
No	1.00 (ref)	1.01 (0.78, 1.31)	0.87 (0.45, 1.66)	
Drinking past year				0.723
Yes	1.00 (ref)	1.09 (0.75, 1.59)	0.33 (0.08, 1.36)	
No	1.00 (ref)	0.99 (0.75, 1.30)	1.11 (0.58, 2.13)	
Physical activity				0.748
Low	1.00 (ref)	1.09 (0.76, 1.58)	0.53 (0.16, 1.69)	
Medium	1.00 (ref)	1.00 (0.69, 1.45)	0.87 (0.40, 1.92)	
High	1.00 (ref)	1.08 (0.71, 1.65)	0.66 (0.16, 2.69)	
Urbanization				0.440
Low	1.00 (ref)	1.10 (0.72, 1.70)	0.73 (0.18, 2.99)	
Medium	1.00 (ref)	0.85 (0.57, 1.25)	0.75 (0.23, 2.40)	
High	1.00 (ref)	1.27 (0.89, 1.81)	0.81 (0.37, 1.78)	
BMI（kg/m^2）				0.203
< 18.5	1.00 (ref)	0.90 (0.30, 2.70)	0.35 (0.04, 3.09)	
18.5–23.9	1.00 (ref)	0.99 (0.73, 1.35)	0.86 (0.42, 1.77)	
≥ 24.0	1.00 (ref)	1.15 (0.83, 1.60)	0.69 (0.25, 1.92)	

* $P < 0.05$, ** $P < 0.01$, *** $P < 0.001$.

Table S2. Stratified analyses of free sugars intake with hypertriglyceridemia

	Intake of free sugars (g/d)			*P* for Interaction
	< 5	5–20	> 20	
Sex				0.978
Men	1.00 (ref)	0.92 (0.70, 1.23)	1.08 (0.61, 1.91)	
Women	1.00 (ref)	1.07 (0.82, 1.39)	0.75 (0.36, 1.54)	
Age				0.808
18–44	1.00 (ref)	0.99 (0.74, 1.32)	1.06 (0.53, 2.11)	
45–59	1.00 (ref)	1.08 (0.80, 1.45)	0.89 (0.43, 1.84)	
≥ 60	1.00 (ref)	0.79 (0.48, 1.31)	0.79 (0.29, 2.14)	
Education level				0.247
Junior high school or below	1.00 (ref)	0.97 (0.76, 1.23)	0.79 (0.37, 1.68)	
Senior high school or above	1.00 (ref)	1.00 (0.72, 1.39)	1.00 (0.57, 1.77)	
Place of residence				0.402
Urban areas	1.00 (ref)	1.05 (0.78, 1.42)	1.16 (0.68, 1.96)	
Rural areas	1.00 (ref)	0.96 (0.75, 1.24)	0.72 (0.30, 1.75)	
Region of residence				0.327
Northern regions	1.00 (ref)	1.11 (0.81, 1.51)	0.91 (0.44, 1.90)	
Southern regions	1.00 (ref)	0.96 (0.75, 1.22)	1.02 (0.58, 1.78)	
Individual annual income				0.508
Low	1.00 (ref)	0.92 (0.63, 1.34)	2.23 (1.06, 4.68) *	
Medium	1.00 (ref)	0.71 (0.50, 1.02)	0.64 (0.23, 1.75)	
High	1.00 (ref)	1.29 (0.95, 1.75)	0.73 (0.37, 1.42)	
Smoking history				0.974
Yes	1.00 (ref)	0.88 (0.62, 1.26)	1.34 (0.64, 2.83)	
No	1.00 (ref)	1.05 (0.83, 1.32)	0.85 (0.49, 1.49)	
Drinking past year				0.381
Yes	1.00 (ref)	0.98 (0.71, 1.36)	0.60 (0.22, 1.65)	
No	1.00 (ref)	1.01 (0.80, 1.28)	1.20 (0.73, 1.97)	
Physical activity				0.159
Low	1.00 (ref)	1.25 (0.91, 1.72)	1.26 (0.65, 2.42)	
Medium	1.00 (ref)	0.76 (0.55, 1.07)	0.77 (0.37, 1.58)	
High	1.00 (ref)	1.01 (0.70, 1.44)	0.62 (0.19, 2.00)	
Urbanization				0.772
Low	1.00 (ref)	0.93 (0.62, 1.41)	0.40 (0.06, 2.91)	
Medium	1.00 (ref)	1.07 (0.78, 1.48)	1.22 (0.53, 2.83)	
High	1.00 (ref)	0.91 (0.67, 1.23)	0.88 (0.51, 1.53)	
BMI（kg/m^2）				0.090
< 18.5	1.00 (ref)	1.95 (0.65, 5.86)	1.84 (0.21, 16.12)	
18.5–23.9	1.00 (ref)	0.94 (0.71, 1.23)	1.34 (0.81, 2.22)	
≥ 24.0	1.00 (ref)	0.99 (0.75, 1.30)	0.32 (0.10, 1.00)	

* $P < 0.05$, ** $P < 0.01$, *** $P < 0.001$.

Table S3. Stratified analyses of free sugars intake with hypo-HDL-cholesterolemia

	Intake of free sugars (g/d)			*P* for Interaction
	< 5	5–20	> 20	
Sex				0.561
Men	1.00 (ref)	1.27 (0.98, 1.65)	1.70 (1.04, 2.77) *	
Women	1.00 (ref)	1.28 (0.97, 1.67)	2.12 (1.26, 3.57) **	
Age				0.392
18–44	1.00 (ref)	1.53 (1.16, 2.03) **	2.50 (1.51, 4.15) **	
45–59	1.00 (ref)	1.09 (0.81, 1.48)	1.26 (0.63, 2.52)	
≥ 60	1.00 (ref)	1.29 (0.80, 2.07)	2.04 (0.95, 4.38)	
Education level				0.390
Junior high school or below	1.00 (ref)	1.23 (0.98, 1.54)	1.87 (1.13, 3.07) *	
Senior high school or above	1.00 (ref)	1.35 (0.97, 1.87)	1.93 (1.14, 3.29) *	
Place of residence				0.873
Urban areas	1.00 (ref)	1.30 (0.96, 1.77)	1.81 (1.13, 2.91) *	
Rural areas	1.00 (ref)	1.23 (0.97, 1.56)	2.18 (1.24, 3.83) **	
Region of residence				0.076
Northern regions	1.00 (ref)	1.29 (0.94, 1.76)	3.16 (1.92, 5.18) ***	
Southern regions	1.00 (ref)	1.30 (1.03, 1.64) *	1.28 (0.76, 2.16)	
Individual annual income				0.348
Low	1.00 (ref)	1.18 (0.84, 1.64)	2.34 (1.21, 4.50) *	
Medium	1.00 (ref)	1.26 (0.91, 1.74)	1.56 (0.75, 3.24)	
High	1.00 (ref)	1.43 (1.03, 2.00) *	1.78 (1.03, 3.07) *	
Smoking history				0.190
Yes	1.00 (ref)	1.06 (0.77, 1.47)	1.67 (0.88, 3.17)	
No	1.00 (ref)	1.43 (1.14, 1.81) **	2.00 (1.30, 3.09) **	
Drinking past year				0.720
Yes	1.00 (ref)	1.37 (1.02, 1.84) *	1.87 (1.06, 3.30) *	
No	1.00 (ref)	1.22 (0.96, 1.56)	1.97 (1.25, 3.13) **	
Physical activity				0.982
Low	1.00 (ref)	1.20 (0.86, 1.66)	1.49 (0.76, 2.91)	
Medium	1.00 (ref)	1.58 (1.17, 2.15) **	2.69 (1.61, 4.51) ***	
High	1.00 (ref)	1.04 (0.73, 1.49)	1.52 (0.69, 3.34)	
Urbanization				0.840
Low	1.00 (ref)	1.24 (0.85, 1.82)	2.64 (1.28, 5.47) **	
Medium	1.00 (ref)	1.19 (0.85, 1.67)	2.32 (1.05, 5.11) *	
High	1.00 (ref)	1.33 (1.00, 1.78)	1.54 (0.95, 2.51)	
BMI（kg/m^2）				0.805
< 18.5	1.00 (ref)	1.35 (0.39, 4.70)	3.55 (0.56, 22.59)	
18.5–23.9	1.00 (ref)	1.40 (1.08, 1.81) *	1.61 (0.97, 2.65)	
≥ 24.0	1.00 (ref)	1.19 (0.90, 1.56)	2.14 (1.24, 3.67) **	

* $P < 0.05$, ** $P < 0.01$, *** $P < 0.001$.

Table S4. Stratified analyses of free sugars intake with hyper-LDL-cholesterolemia

	Intake of free sugars (g/d)			*P* for Interaction
	< 5	5–20	> 20	
Sex				0.100
Men	1.00 (ref)	1.04 (0.77, 1.41)	1.40 (0.79, 2.48)	
Women	1.00 (ref)	1.47 (1.14, 1.89) **	1.56 (0.86, 2.84)	
Age				0.957
18–44	1.00 (ref)	1.24 (0.88, 1.75)	1.54 (0.77, 3.11)	
45–59	1.00 (ref)	1.21 (0.92, 1.60)	1.67 (0.91, 3.06)	
≥60	1.00 (ref)	1.52 (1.01, 2.29) *	1.66 (0.63, 4.37)	
Education level				0.318
Junior high school or below	1.00 (ref)	1.13 (0.90, 1.43)	1.74 (1.02, 2.97) *	
Senior high school or above	1.00 (ref)	1.67 (1.18, 2.37) **	1.45 (0.75, 2.81)	
Place of residence				0.182
Urban areas	1.00 (ref)	1.44 (1.03, 2.03) *	1.94 (1.13, 3.31) *	
Rural areas	1.00 (ref)	1.22 (0.97, 1.55)	1.14 (0.56, 2.32)	
Region of residence				0.963
Northern regions	1.00 (ref)	1.36 (1.01, 1.82) *	1.15 (0.58, 2.30)	
Southern regions	1.00 (ref)	1.22 (0.94, 1.57)	1.87 (1.12, 3.13) *	
Individual annual income				0.179
Low	1.00 (ref)	1.51 (1.08, 2.10) *	0.94 (0.34, 2.59)	
Medium	1.00 (ref)	0.85 (0.59, 1.22)	1.37 (0.63, 2.99)	
High	1.00 (ref)	1.52 (1.10, 2.10) *	1.85 (1.03, 3.32) *	
Smoking history				0.835
Yes	1.00 (ref)	1.00 (0.68, 1.47)	1.71 (0.87, 3.35)	
No	1.00 (ref)	1.35 (1.08, 1.68) **	1.34 (0.80, 2.27)	
Drinking past year				0.996
Yes	1.00 (ref)	1.27 (0.90, 1.80)	1.43 (0.70, 2.90)	
No	1.00 (ref)	1.26 (1.00, 1.58)	1.66 (1.00, 2.77)	
Physical activity				0.090
Low	1.00 (ref)	1.39 (1.01, 1.93) *	1.53 (0.75, 3.11)	
Medium	1.00 (ref)	1.51 (1.10, 2.07) *	1.58 (0.83, 3.04)	
High	1.00 (ref)	0.97 (0.66, 1.41)	1.37 (0.59, 3.15)	
Urbanization				0.230
Low	1.00 (ref)	1.18 (0.78, 1.78)	1.01 (0.32, 3.22)	
Medium	1.00 (ref)	1.34 (0.97, 1.84)	1.15 (0.46, 2.87)	
High	1.00 (ref)	1.38 (1.01, 1.88) *	1.94 (1.15, 3.29) *	
BMI（kg/m^2）				0.523
< 18.5	1.00 (ref)	1.88 (0.81, 4.36)	0.88 (0.11, 7.07)	
18.5–23.9	1.00 (ref)	1.23 (0.94, 1.60)	1.44 (0.85, 2.44)	
≥ 24.0	1.00 (ref)	1.28 (0.96, 1.72)	1.53 (0.76, 3.10)	

* $P < 0.05$, ** $P < 0.01$, *** $P < 0.001$.
